# Targeting CA2 Perineuronal Nets Restores Recognition Memory and Theta Oscillations in Aged Mice

**DOI:** 10.1111/acel.70139

**Published:** 2025-06-16

**Authors:** Sonam Fathima Mehak, Apoorva Bettagere Shivakumar, Feyba Jijimon, Amritanshu Gupta, Vikram Gopalakrishna Pillai, Gireesh Gangadharan

**Affiliations:** ^1^ Department of Ageing Research, Manipal School of Life Sciences Manipal Academy of Higher Education Manipal Karnataka India; ^2^ Department of Biomedical Engineering, Manipal Institute of Technology Manipal Academy of Higher Education Manipal Karnataka India; ^3^ Department of Pathology and Laboratory Medicine University of Kansas Medical Center Kansas City Kansas USA

**Keywords:** aging, extracellular matrix (ECM), perineuronal nets (PNN), recognition memory, theta oscillations

## Abstract

Remembering familiar versus novel stimuli is fundamental to survival, but it is compromised in several neurodegenerative disorders where aging is a key factor. Although the components of the extracellular matrix (ECM) have been suggested to be implicated in memory maintenance, the mechanistic and behavioral roles of ECM during the aging process remain unclear. Here, we employed an accelerated mouse model of aging to elucidate the causal link between ECM dynamics and recognition memory during aging. Aged mice exhibited impaired social and non‐social recognition memory, accompanied by increased intensity of perineuronal nets (PNNs), specialized ECM structures in the hippocampal dorsal CA2 (dCA2). A reduction in the power of theta oscillations (3–7 Hz) in the dCA2 of aged mice was also observed. Notably, selective degradation of PNNs in the dCA2 using chondroitinase ABC (ChABC) rescued recognition memory deficits and restored theta oscillations. Together, our findings identify abnormal PNN in the CA2 as a critical factor for age‐related deficits in hippocampal‐dependent recognition memory and network rhythmicity. These insights raise the possibility that targeting CA2 PNNs could facilitate the development of diagnostic and therapeutic strategies to address age‐associated cognitive frailty.

## Graphical abstract

Targeting CA2 perineuronal nets restores social/non‐social recognition memory and theta oscillations in aged mice: Aged mice displayed deficits in both social and object recognition memory, which were accompanied by enhanced perineuronal nets (PNNs) density and reduced theta power in the dCA2 region. Reducing CA2 PNNs to control levels using chondroitinase ABC (chABC) in aged mice rescued the memory impairments and enhanced hippocampal theta power.

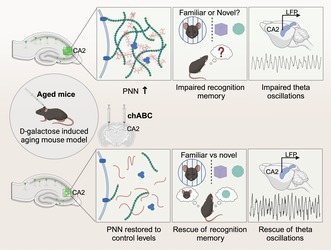

## 1 Introduction

Aging is a significant risk factor for cognitive decline, with deficits in hippocampal‐dependent behaviors being a prominent feature of age‐related neurological disorders such as Alzheimer's disease (AD) and other dementias (Koh et al. 2014). Recognition memory, the ability to identify and distinguish between previously encountered social and non‐social stimuli and novel ones, is a fundamental cognitive process vulnerable to aging and relies heavily on intact hippocampal circuitry (Arias‐Cavieres et al. 2017). Within the hippocampus, the CA2 area is known for its specialized roles in memory processes, owing to its central location, extensive connectivity, and unique molecular signatures (Middleton and McHugh 2020). The role of dorsal CA2 (dCA2) in social recognition memory is well established (Hitti and Siegelbaum 2014; Shivakumar et al. 2024), and emerging evidence suggests that its functions extend beyond social contexts, encompassing broader roles in recognition memory tasks and novelty detection (Lehr et al. 2021). CA2 place cells exhibit remapping not only in response to social stimuli but also when a novel object is introduced into a familiar environment (Wintzer et al. 2014). Further, disrupting CA3a/CA2 to CA1 projections impairs object recognition (Raam et al. 2017), whereas enhancing CA3‐CA2 plasticity improves object recognition and spatial learning (Lee et al. 2010). Although several unique properties of CA2 neurons are known to change with aging, including alterations in parvalbumin (PV) interneurons and pyramidal neurons (Piskorowski et al. 2016), the precise mechanisms by which CA2 contributes to hippocampal‐dependent memory dysfunction in aging remain largely unexplored.

At the molecular level, specialized extracellular matrix (ECM) structures called perineuronal nets (PNNs) are crucial for preserving the structural and functional integrity of hippocampal neurons (Carulli et al. 2021). PNNs, predominantly composed of chondroitin sulfate proteoglycans (CSPGs) that envelop the soma and proximal dendrites of specific neuronal classes, regulate plasticity through the sulfated chondroitin sulfate glycosaminoglycan (CS‐GAG) chains of the CSPGs (van Spijker and Kwok 2017). The CA2 area, in particular, is uniquely enriched with PNNs in contrast to their sparser distribution in other subfields (Lensjø et al. 2017). Further, CA2 PNNs also enclose excitatory pyramidal neurons, unlike other hippocampal regions where PNNs predominantly surround inhibitory interneurons (Domínguez et al. 2019). This unique association suggests a specialized function of these ECM structures in modulating neuronal excitability, potentially contributing to the maintenance of excitatory/inhibitory (E/I) balance. Functionally, CA2 is also critical for orchestrating hippocampal network dynamics, including theta oscillations‐ rhythmic neural oscillatory patterns in the 3–12 Hz range, which are essential for synchronizing hippocampal activity during memory encoding and retrieval (Oliva et al. 2023). Emerging evidence further underscores the importance of theta oscillations in social recognition memory (Shivakumar et al. 2025). Further, aberrant PNN expression has been reported across different brain regions during aging and in neurological conditions like AD, depression, and schizophrenia (Dong et al. 2023; Wen et al. 2018). Despite these insights, the precise involvement of CA2 PNNs in aging has received limited investigation.

We hypothesized that CA2 PNNs may serve as a vital link between theta oscillations and hippocampal‐dependent behaviors, which we explored using an accelerated aging mouse model induced through chronic D‐galactose administration. Aged mice displayed deficits in both social and object recognition memory, which were accompanied by enhanced PNN density and reduced theta power in the dCA2 region. Strikingly, reducing CA2 PNNs to control levels using chondroitinase ABC (chABC) in aged mice rescued the memory impairments and enhanced hippocampal theta power. Together, our findings reveal that CA2 PNN contributes to both social and non‐social recognition memory deficits in aging via modulating hippocampal rhythmicity.

## 2 Methodology

### 2.1 Animals

Eight weeks old C57BL/6 male mice, weighing 25–30 g, were used throughout the study unless mentioned otherwise. Male mice were chosen due to the sex bias in social behavior paradigms, particularly in the three‐chamber test, where female mice typically show lower social exploratory behavior and greater variability due to hormonal fluctuations affecting exploration and recognition behaviors (Cordeira et al. 2018; Krueger‐Burg et al. 2016). The animals were housed in groups of four in a pathogen‐free environment, maintained at a temperature of 22°C ± 2°C with a light/dark cycle of 12‐h (light onset at 6:30 AM), and had unrestricted access to food and water. All procedures and handling were performed as per the Institutional Animal Ethical Committee, Kasturba Medical College, Manipal Academy of Higher Education, Manipal, India (IAEC/KMC/17/2022, dated February 26, 2022).

### 2.2 D‐Galactose Mouse Model of Accelerated Aging

**FIGURE 1:**
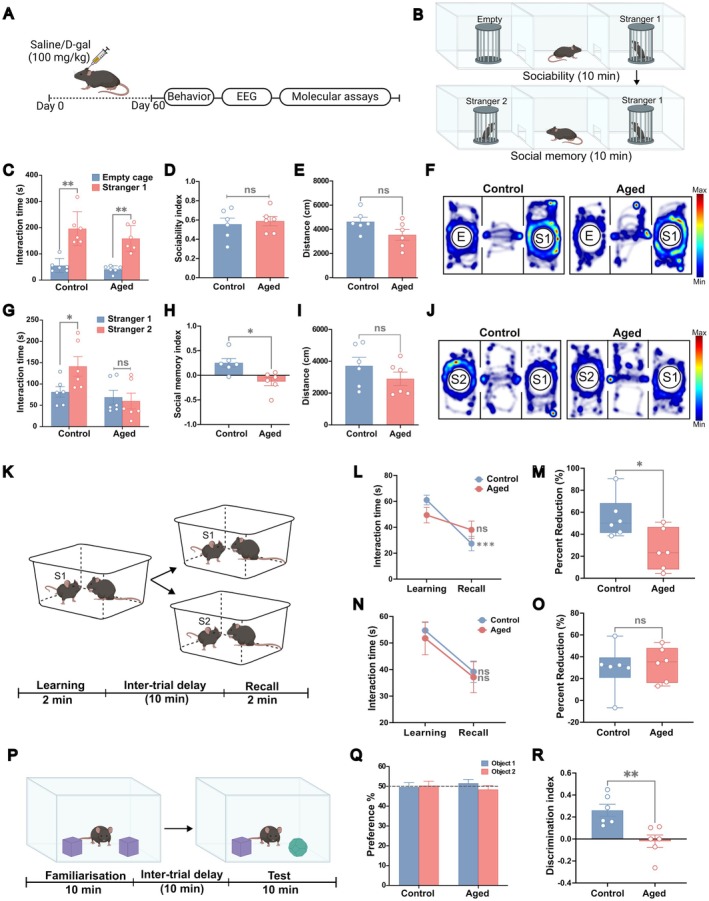
Impaired social and object recognition memory in aged mice. (A) Experimental timeline: Behavioral, electrophysiological, and molecular analyses were performed 60 days post D‐galactose/saline injections. (B) Schematic of the three‐chamber social memory test. (C) Interaction during sociability phase: Both aged and control mice exhibited intact sociability, preferring stranger 1 (S1) over empty cage. (D) Sociability index showed no significant difference between the groups. (E) Total distance traveled by subject mice during sociability. (F) Representative heatmaps of the sociability phase. (G) Interaction during social memory phase: Control, but not aged mice, spent significantly more time interacting with Stranger 2 (S2), showing impaired social memory in aging. (H) Social memory index of aged mice was significantly lower. (I) Total distance traveled by subject mice during social memory phase. (J) Representative heatmaps of social memory phase. (K) Schematics of the two‐trial test with familiar (S1) and novel (S2) juvenile stimuli. (L) Interaction during learning and recall phases with familiar stimulus: Only control mice showed reduced interaction during recall, indicating impaired social memory in aged mice. (M) Percentage reduction in interaction time from learning to recall phase was significantly lower in aged mice. (N) Interaction during learning and recall with novel stimulus: Both control and aged mice showed reduction in interaction during recall phase, showing intact social motivation. (O) Percentage reduction in interaction from learning to recall phase was similar among the two groups. (P) Schematics of the object recognition test. (Q) Percentage preference for object 1 and 2 during familiarization phase. (R) Discrimination index was significantly reduced in aged mice. *n* = 6 per group, all data represented are mean ± SEM; ns, non‐significant, *p < 0.05, **p < 0.01, ***p < 0.001, Student's *t*‐test and two‐way ANOVA with Sidak's multiple comparison test.

The D‐galactose model offers a time‐efficient, controlled approach to studying brain aging, replicating key features like oxidative stress, neuroinflammation, and synaptic changes. Unlike natural aging, which varies due to genetic and environmental factors, this model ensures reproducibility while avoiding significant motor dysfunction, making it ideal for behavioral assessment (Hou et al. 2019; Li et al. 2019). The mice were randomly assigned to two groups for control saline (*n* = 12) and D‐galactose (*n* = 18) administration. Eight‐week‐old mice were given subcutaneous injections of D‐galactose (D‐gal; G0750, Sigma‐Aldrich, USA) at a dosage of 100 mg/kg body weight (dissolved in 0.9% saline to a stock concentration of 20 mg/mL) for 60 consecutive days (Wei et al. 2005). The control group received an equivalent volume of saline administered subcutaneously in the same manner (Figure 1A). All subsequent experiments were performed using the same batch of the D‐gal‐treated mice, hereafter referred to as aged mice. Detailed experimental timeline and sample sizes are provided in Figure S1.

### 2.3 Behavioral Assays

All behavioral assays were conducted during the light phase, between 9 AM and 5 PM. Mice were acclimatized to the researcher and experimental room 2 days prior to the testing and transferred to the room 60 min before experiments began. The behavioral tests were performed in the following sequence: Open field test (OFT) (Supplementary methods), novel object recognition test (NORT), three‐chamber test (TCT), two‐trial (2T) social memory test, and elevated plus maze (EPM) (Supplementary methods). All stranger mice used in the social memory experiments were novel, juvenile male mice (6 weeks old) with no previous exposure to the subject mouse in any earlier experiments. The experiments were videotaped, and the behavioral tracking system EthoVision XT 15 (Noldus, Netherlands) or manual analysis was used for analysis.

#### 2.3.1 Three‐Chamber Test

The three‐chamber test for sociability and social recognition memory was performed as previously described (Figure 1B) (Moy et al. 2004). The apparatus consisted of a transparent Plexiglass (60 × 40 × 40 cm) divided into three chambers with retractable doorways in the partition walls allowing access between chambers. During the initial habituation phase, the mice were given 10 min to freely explore each of the three compartments. Immediately after this, an unfamiliar male juvenile mouse (stranger 1, S1) that had no previous interaction with the subject mouse, was enclosed in a wire cage and placed inside either of the side chambers. The opposite chamber contained a similar empty wire cage, allowing the subject mouse to explore for 10 min freely (sociability phase). At the end of the sociability phase, the mice were placed back in their home cages. Following an inter‐trial delay of 10 min, another novel, unfamiliar male juvenile (stranger 2, S2) mouse was introduced into the previously empty wire cage. The subject mouse was again given 10 min to freely explore all three chambers, with a free choice between the familiar (S1) and novel (S2) mouse (social memory phase). The interaction time (s) with the stranger mice and empty wire cage during both phases and the total distance traveled were measured. The preference index for sociability ($I_{S}=\frac{\text{Time with stranger}\;1-\text{time with empty cage}}{\text{Total exploration time}}$) and social memory ($I_{\mathrm{SM}}=\frac{\text{Time with stranger}\;2-\text{time with stranger}\;1}{\text{Total exploration time}}$) were calculated accordingly.

#### 2.3.2 Two‐Trial Social Memory Test

Social recognition memory was further assessed using the two‐trial social memory test as previously described by Kogan et al. (2000) (Figure 1K). Briefly, the mice were initially acclimated to a clean home cage (22 × 15 × 15 cm) for 15 min. A stranger male juvenile mouse (S1) was then introduced into the cage for an initial interaction trial (learning session) of 2 min. Following an intertrial delay of 10 min, the same juvenile mouse (familiar) was re‐introduced to the cage for a 2‐min test trial (recall session). In a parallel experiment, another set of mice from the aged and control groups underwent the same experimental procedures, except that another novel juvenile mouse (S2) was introduced during the recall session. Behaviors classified as social investigation included direct interaction with the juvenile, such as examining body surfaces through grooming, licking, pawing, and sniffing, as well as closely following the juvenile within a 1 cm distance. The interaction time (s) with the juvenile in each session was measured and the percentage reduction in interaction from the learning to recall sessions was calculated as follows:

$$\%\text{reduction}=\left(\frac{\text{Exploration time during learning}-\text{exploration time during recall}}{\text{Exploration time during learning}}\right)\times 100$$

#### 2.3.3 Novel Object Recognition Test

Object recognition memory was evaluated using the NOR test, adapted from Lueptow (2017) with slight alterations (Figure 1P). Mice were tested in an open arena (40 × 40 × 40 cm) across three phases. After acclimatizing the mice for 10 min to the open field environment (habituation phase), two identical objects were positioned equidistantly, and the mouse was given 10 min to explore (familiarization phase). Following an inter‐trial delay of 10 min, the mice were returned to the arena, where one object was swapped for a novel one, and allowed exploration for an additional 10 min (test phase). Object recognition was quantified as the number of approaches to each object, represented as the discrimination index (DI) with the following formula:

$$\mathrm{DI}=\frac{\mathrm{No}.\text{of approaches to novel object}-\mathrm{no}.\text{of approaches to familiar object}}{\text{Total}\;\mathrm{no}.\;\text{of approaches}}$$

### 2.4 Stereotaxic Surgery

Stereotaxic surgery was performed to implant electrodes for local field potential (LFP) recording and chABC administration. All surgical procedures on mice were performed under intraperitoneal (i.p.) anesthesia using a ketamine (100 mg/kg) and xylazine (10 mg/kg) cocktail. The mice were positioned carefully in a stereotaxic apparatus (Stoelting Co., USA), with the head leveled using bregma and lambda as reference points.

#### 2.4.1 In Vivo LFP Recording and Data Analysis

Cranial openings were created using dental drills for electrode implantation, and a Teflon‐coated tungsten electrode (796500, A‐M Systems, USA) was unilaterally inserted into the dCA2 on the right hemisphere (from bregma: −1.6 anteroposterior, ±1.6 mediolateral, and −1.7 dorsoventral) (Paxinos and Franklin 2019). Grounding was achieved by placing a ground electrode over the cerebellum, with a reference electrode implanted in the frontal lobe. In the chABC group, electrodes were inserted into the right hemisphere immediately after bilateral injection of chABC to the same coordinates. Post a recovery period of 7 days, Hippocampal LFPs were recorded from the control, aged, and chABC groups under urethane anesthesia (1.2 g/kg, i.p.), following established protocols (Gangadharan et al. 2016; Shivakumar et al. 2025), using an RHS stim/recording system (Intan Technologies, USA). The electrode placement was confirmed post‐mortem through histological analysis using Nissl staining, following standard protocols (Gangadharan et al. 2016).

Offline analysis of LFP data was conducted in MATLAB (R2023b) using both built‐in functions and custom scripts, adhering to established methodologies (Gangadharan et al. 2016; Shivakumar et al. 2025). Signals, sampled at 1000 Hz, were preprocessed using a second‐order Butterworth bandpass filter (1–30 Hz). The power spectral density (PSD) was calculated using the Welch method, where the signal was segmented with overlapping windows, each subjected to a Hamming window, and the periodogram was computed for each segment. PSD plots emphasized the theta band (3–7 Hz), with overlaid runs enabling comparative analysis across trials. For mean theta power analysis, significant theta events were detected using the Short‐Time Fourier Transform (STFT), performed with a 4‐s window, 50% overlap, and 1024 FFT points. Theta power (3–7 Hz) was integrated within each window, and events were defined as instances where theta power exceeded the mean plus one standard deviation. The mean theta power of these events was calculated and recorded for each trial. Similarly, for theta amplitude analysis, preprocessing included the Hilbert Transform to compute the amplitude envelope of theta events. The mean amplitude of these theta events was then quantified for each trial.

#### 2.4.2 chABC Administration

To investigate whether the increased PNN density observed in aged mice contributed causally to the behavioral and electrophysiological changes, we used chABC (C3667, Sigma‐Aldrich, USA), an enzyme that temporarily degrade PNNs by cleaving the chondroitin sulfate chains critical for PNN structural integrity (Chu et al. 2018). Aged mice received a single bilateral injection of 1 μL chABC (dissolved in PBS with 0.1% BSA to a final concentration of 25 U/mL) per hemisphere (Végh et al. 2014; Yang et al. 2015) to the dCA2 (from bregma, AP −1.6 mm, ML ±1.6 mm, and DV −1.7 mm). The injections were carried out at a flow rate of 0.3 μL/min with a NanoFil 33 g blunt needle (World Precision Instruments, USA) connected to a Hamilton syringe (25 μL, 802N, 22s ga/51 mm; Hamilton, Switzerland) that was controlled by an infusion pump (Braintree scientific, USA). The needle was held in place for an additional 5 min to ensure proper diffusion. For the behavioral experiments, the control saline group received an equivalent volume of PBS (sham) similarly. Post‐surgery, the health of the mice was monitored, and they were given a 5‐day recovery period. Electrophysiological and behavioral studies were then conducted on separate cohorts of mice to ensure optimal timing, as the effects of a single chABC injection last for approximately 10 days (Figure 4A).

### 2.5 Immunohistochemistry (IHC)

Mice were chosen at random after the behavioral and electrophysiological experiments for IHC analysis, adapted with slight modifications from Horii‐Hayashi et al. (2015). To determine whether naturally aged mice exhibit similar expression patterns, we performed IHC on hippocampal sections from naturally aged mice, comparing young (2‐month‐old) and aged (22‐month‐old) cohorts (*n* = 1 per group). Briefly, transcardial perfusion was performed with phosphate‐buffered saline (PBS, pH 7.4), followed by 4% paraformaldehyde (PFA) in 0.1 M phosphate buffer (pH 7.4) under ketamine (100 mg/kg)/xylazine (10 mg/kg) anesthesia. The extracted brains were post‐fixed in PFA for 24 h and then transferred to PBS until sectioning. Coronal sections (30 μM thickness) of the entire dorsal hippocampus were collected using a vibratome (5100 mz, Campden instruments, UK). After 5 min of PBS rinsing, the chosen sections were incubated for 30 min in PBS containing 0.1% Triton X‐100 (PBST). Following a 5‐min PBS rinse, the sections were pre‐blocked in PBST containing 2% bovine serum albumin for 1 h. The sections were rinsed again and incubated with biotinylated *Wisteria floribunda* agglutinin (WFA, 1:500, B‐1355‐2, Vector Laboratories, USA) at 4^o^ overnight. After three PBS washes, the sections were incubated for 2 h at room temperature in the dark with Alexa fluor 488‐conjugated avidin (1:1000, A21370, Invitrogen, USA). Post rinsing, sections were mounted onto slides and coverslipped using Vectashield containing 4′,6‐diamidino‐2‐phenylindole dihydrochloride (DAPI; Vector Laboratories). Images were captured using a BX53 fluorescent microscope (Olympus, Tokyo, Japan) with 4× and 10× objective lenses. All sections were processed and imaged simultaneously, with consistent exposure settings maintained across all sections.

### 2.6 Quantification of Optical Intensity and PNN Density

WFA optical intensity in the dCA2 was measured from three anatomically matched sections per animal using ImageJ (NIH, USA). After background subtraction (rolling ball radius = 50 pixels), the ROI function was used to draw a perimeter around the CA2 pyramidal cell layer and the threshold tool was used to highlight only the ring‐like PNN structures. The intensity was determined by multiplying the maximum mean gray value by the percent area. The resulting maximum intensity values for WFA were then averaged per section for each animal, and values were normalized to that of the control. For the purpose of quantifying the density (PNNs/mm^2^), the number of WFA‐positive PNNs within the defined CA2 ROI were counted using the cell counter plug‐in. The total number of PNNs was then divided by the measured area to obtain PNN density.

### 2.7 Statistics

All results represented are mean ± SEM. The data were evaluated by appropriate statistical tests (Student's *t*‐test, one‐way and two‐way ANOVA with Sidak's multiple comparison test/post hoc Tukey–Kramer test) using GraphPad Prism 9 (v9.5.1).

## 3 Results

### 3.1 Aged Mice Exhibit Deficits in Hippocampal‐Dependent Recognition Memory

#### 3.1.1 Impaired Social Recognition Memory With Intact Sociability in Aged Mice

To assess social recognition memory, we performed the three‐chamber and two‐trial social memory tests. Our results reveal that aged mice show intact sociability in the three‐chamber test, with both the control and aged mice spending significantly more time interacting with the stimulus mouse (stranger 1) (Figure 1C,F) (interaction time (s), control, stranger 1: 196.40 ± 26.47, empty cage: 55.45 ± 10.95, *p* = 0.003, *t*(5) = 5.17; aged, stranger 1: 158.70 ± 20.12, empty cage: 40.79 ± 5.66; *p* = 0.001, *t*(5) = 6.84, paired *t*‐test). The sociability index, calculated as the ratio of interaction time with stranger 1 to the empty cage, showed no statistical difference between the groups (Figure 1D) (sociability index, control: 0.56 ± 0.06, aged: 0.59 ± 0.05; *p* = 0.697, *t*(10) = 0.40, unpaired *t*‐test). However, in the social memory phase, control mice demonstrated a strong preference for the novel stranger (stranger 2), while aged mice spent a similar duration investigating familiar (stranger 1) and novel stimuli (Figure 1G,J) (interaction time (s), control, stranger 1: 85.18 ± 13.84, stranger 2: 149.20 ± 20.82, *p* = 0.035, *t*(5) = 2.87; aged, stranger 1: 69.06 ± 16.31, stranger 2: 60.14 ± 18.44; *p* = 0.124, *t*(5) = 1.85, paired *t*‐test). Further, aged mice exhibited reduced social memory index, indicating impaired social recognition memory (Figure 1H) (social memory index, control: 0.25 ± 0.09, aged: −0.13 ± 0.08; *p* = 0.010, *t*(10) = 3.15, unpaired *t*‐test). The total distance traveled within the chamber during the sociability and social memory phases was similar between the groups (Figure 1E,I) (sociability: distance traveled (cm), control: 4636 ± 357.50, aged: 3543 ± 442.40, *p* = 0.084, *t*(10) = 1.92; social memory: distance traveled (cm), control: 3717 ± 532.00, aged: 2902 ± 421.40, *p* = 0.257, *t*(10) = 1.20 unpaired *t*‐test), suggesting that the observed changes are not secondary to differences in overall activity or impaired locomotion, which are consistent with our open field test (Figure S2A,D). Anxiety levels remained normal in EPM (Figure S2E,G), indicating cognitive decline rather than an attention deficit.

We corroborated these findings using the two‐trial social memory test, in which the subject mouse was introduced to a stranger mouse and re‐exposed to the same mouse (S1) after a 10‐min inter‐trial delay. Consistent with the three‐chamber test, aged mice displayed sociability comparable to control mice during the learning phase (interaction time (s), learning, control, 61.06 ± 3.73, aged: 49.41 ± 5.98, *p* = 0.173, unpaired *t*‐test). The control mice exhibited reduced interaction time from the learning to the recall phase. In contrast, the aged mice displayed no difference in investigation times between the two phases (Figure 1L) (interaction time (s), control, learning: 61.06 ± 3.73, recall: 27.41 ± 5.57, *p* = 0.0008; aged, learning: 49.41 ± 5.98, recall: 38.04 ± 6.73, *p* = 0.307, two‐way ANOVA with Sidak's multiple comparison test). Additionally, the percentage reduction in interaction time between the two phases was markedly lower in aged mice (Figure 1M) (% reduction, control: 55.38 ± 7.71, aged: 25.99 ± 7.66; *p* = 0.022, *t*(10) = 2.71, unpaired *t*‐test).

To rule out the possibility of a potential lack of social motivation, the two‐trial experiment was performed in another set of aged and control mice, with a novel juvenile stranger (S2) being introduced in the recall phase. The control mice did not display any significant difference in the interaction time during the learning and recall phases (interaction time (s), control, learning: 54.73 ± 3.02; recall: 39.16 ± 4.04, *p* = 0.074, two‐way ANOVA with Sidak's multiple comparison test). Similarly, the aged mice showed no significant reduction in social interaction in the recall phase compared to the learning phase (Figure 1N) (interaction time (s), aged, learning: 51.77 ± 6.20; recall: 37.10 ± 5.82, *p* = 0.095, two‐way ANOVA with Sidak's multiple comparison test). Further, the percentage reduction in interaction time was similar between the groups (Figure 1O) (% reduction, control: 29.72 ± 8.57, aged: 33.42 ± 6.43, *p* = 0.738, *t*(10) = 0.34, unpaired *t*‐test). These findings indicate that the observed reduction in the initial two‐trial experiment was specific to memory retention rather than decreased social motivation or recognition of a novel stimulus.

#### 3.1.2 Impaired Object Recognition Memory in Aged Mice

The hippocampus is well‐known for its role in processing contextual details of objects (Manns et al. 2003). Consistent with previous findings (Gao et al. 2021; Sun et al. 2018), our results suggest that aged mice exhibit deficits in NOR. During the familiarization phase, both control and aged mice showed comparable preferences for object 1 and object 2 (Figure 1Q, Figure S2H) (No. of approaches: control, object 1: 49.64 ± 2.22, object 2: 50.36 ± 2.22, *p* = 0.876, *t*(5) = 0.16; aged, object 1: 51.57 ± 1.93, object 2: 48.43 ± 1.93, *p* = 0.452, *t*(5) = 0.82, Interaction time (s): control, object 1: 46.17 ± 3.51, object 2: 53.83 ± 3.51, *p* = 0.20; aged, object 1: 50.26 ± 4.30, object 2: 49.74 ± 3.40, *p* = 0.99, paired *t*‐test). In the test phase that followed, only the control group demonstrated a distinct preference for the novel object, whereas aged mice showed no such preference, as indicated by their reduced discrimination index (Figure 1R, Figure S2I) (DI with number of approaches, control: 0.26 ± 0.05, aged: −0.02 ± 0.06, *p* = 0.005, *t*(10) = 3.58; DI with interaction time, discrimination index with interaction time (s), control: 0.35 ± 0.08, aged: −0.03 ± 0.11, *p* = 0.038, unpaired *t*‐test).

### 3.2 WFA Intensity and PNN Density Are Enhanced in the dCA2 of Aged Mice

**FIGURE 2:**
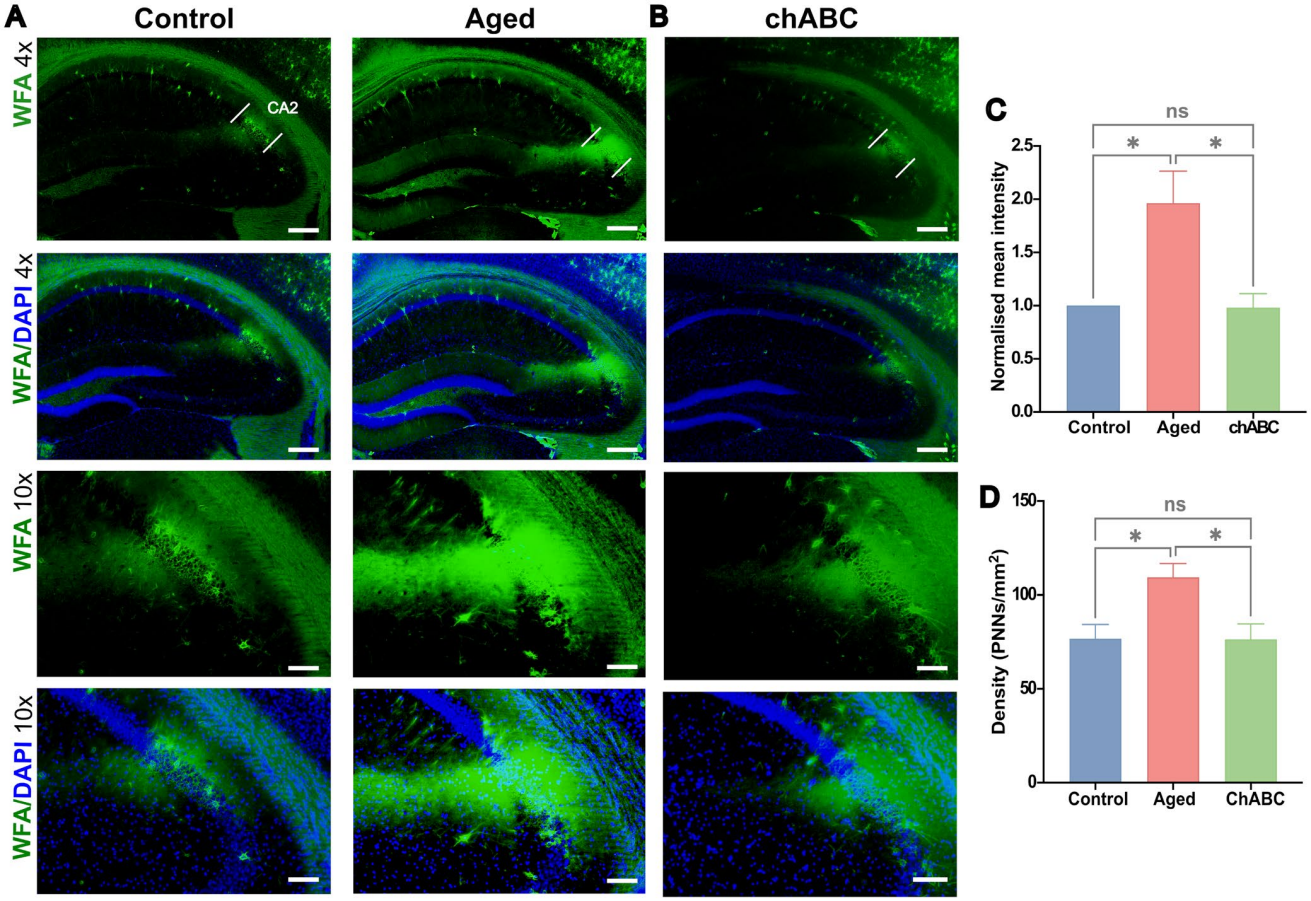
Aged mice show increased WFA fluorescence intensity and PNN density in the dCA2. (A) Representative images of the hippocampus labeled with the PNN marker WFA (green) in control and aged mice. Aged mice showed increased WFA intensity in the CA2, as depicted in 10× magnification images. (B) chABC administration restored PNN to control levels in aged mice. (C) Normalized mean intensity of WFA fluorescence in control, aged, and chABC mice. (D) PNN density in control, aged, and chABC mice. Scale bars = 200 μM (4×); 100 μM (10×). *n* = 3 per group, all data represented are mean ± SEM; ns, non‐significant, *p < 0.05, one‐way ANOVA with post hoc Tukey–Kramer test.

We next investigated whether the PNNs in the hippocampus undergo alterations with aging and employed lectin‐based WFA staining to visualize and assess PNN density. We observed increased WFA staining intensity as well as the PNN density in area CA2 of aged mice in comparison to the control (Figure 2A,C,D) (mean intensity: control vs. aged, *p* = 0.040, density (PNNs/mm^2^): control vs. aged, *p* = 0.014, one‐way ANOVA with post hoc Tukey–Kramer test). Consistent with observations in D‐gal‐treated aged mice, we observed that WFA staining showed a similar enhancement exclusively in the CA2 of naturally aged mice (Figure S3A,B). Additionally, no evident difference in WFA staining intensity was observed within the CA1 and CA3 regions (mean intensity CA1: control vs. aged, *p* = 0.201, CA3: control vs. aged, *p* = 0.972, one‐way ANOVA with post hoc Tukey–Kramer test) (Figure S3C,D). These findings raise the possibility that the accumulation of PNNs, specifically in the CA2, may contribute to the impaired recognition memory observed in aged mice.

### 3.3 Aged Mice Exhibit Attenuated Hippocampal Theta Oscillations

**FIGURE 3:**
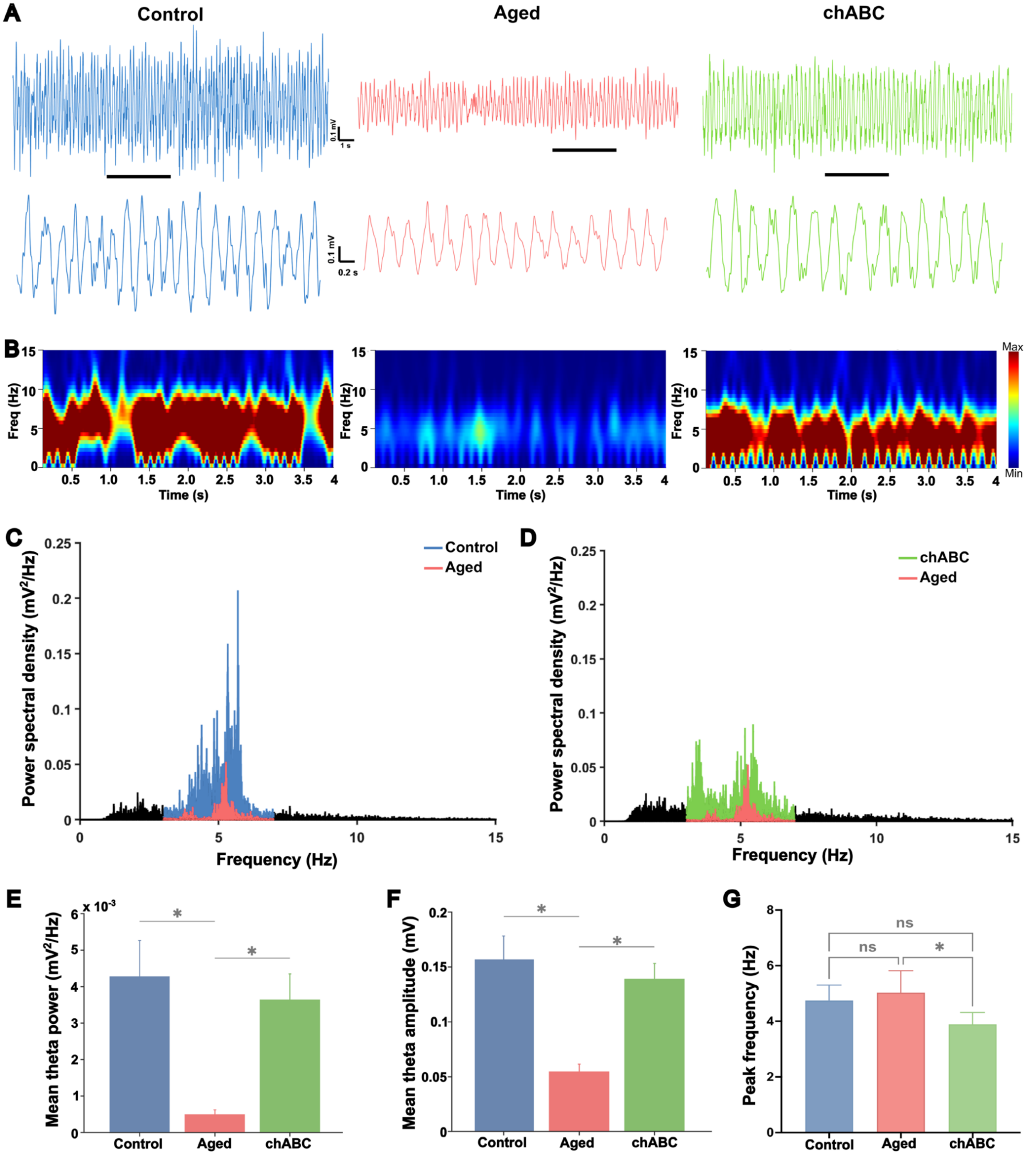
PNN attenuation in the dCA2 rescued theta oscillation in aged mice. (A) Representative LFP traces (20 s and 4 s) and (B) power spectrograms of control, aged, and chABC groups under urethane anesthesia. (C, D) Power spectral density highlight theta frequency (3–7 Hz) in control (blue), aged (red), and chABC (green), illustrating decreased theta power in aged mice and rescue by chABC treatment. Mean theta (E) power and (F) amplitude across groups, showing significant reductions in aged mice, and recovery with chABC. (G) Shift in peak theta frequency in aged mice after treatment with chABC. *n* = 6 per group, all data represented are mean ± SEM; ns, non‐significant, *p < 0.05, one‐way ANOVA with post hoc Tukey–Kramer test.

The observed increase in CA2 PNN density in aged mice may influence neuronal excitability in this region. PV interneurons are closely associated with theta frequency oscillations, demonstrating strong phase‐locking and serving as a source of inhibition that can trigger theta‐resonant membrane oscillations in pyramidal neurons (Stark et al. 2013). Considering that CA2 PNN encapsulates both PV and pyramidal neurons, we measured spontaneous LFP from the dCA2 of the experimental mice under urethane anesthesia, a widely used approach for preserving and isolating theta activity (3–7 Hz) in mice (Mondino et al. 2024; Shivakumar et al. 2025), to investigate potential functional changes. Intermittent theta oscillations were clearly discernible in the LFP recordings from the hippocampal dCA2 area of undisturbed animals following urethane administration. A significant drop in theta power was seen in aged mice compared to the control group, as indicated by power spectral density analysis displaying the distribution of theta power across trials within the 3–7 Hz range (Figure 3A–C). Specifically, the mean power of theta, as well as the amplitude was significantly lower in aged mice in comparison to the control (Figure 3E,F) (Mean theta power, control vs. aged, *p* = 0.0040; mean theta amplitude, control vs. aged, *p* = 0.0007, one‐way ANOVA with post hoc Tukey–Kramer test). The position of the recording electrode in the hippocampal CA2 was confirmed using Nissl staining (Figure S2K).

### 3.4 Degrading Excess CA2 PNN Rescued Memory Deficits in Aged Mice

#### 3.4.1 PNN Depletion Restored Social Recognition Memory Deficits in Aged Mice

**FIGURE 4:**
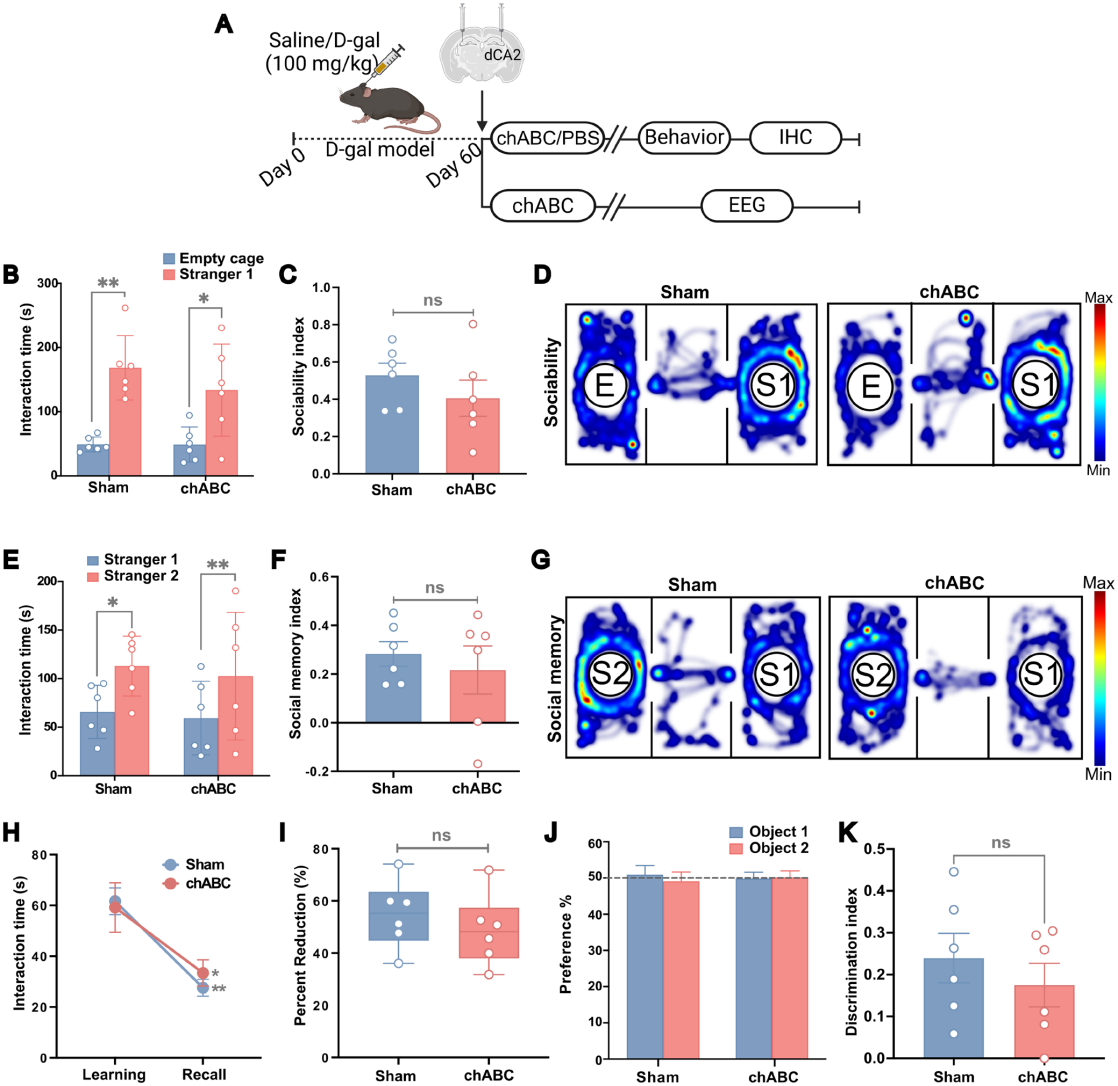
PNN attenuation in dCA2 rescued recognition memory deficits in aged mice. (A) Experimental timeline; control and aged treated mice received PBS (sham) or chABC in CA2, followed by behavioral or electrophysiological experiments, and immunohistochemical validation. (B) Three chamber test; Interaction during sociability phase: Both sham and chABC mice exhibited intact sociability, preferring interaction with stranger 1 over empty cage. (C) Sociability index showed no significant difference between the groups. (D) Representative heatmaps of sociability phase. (E) Interaction during social memory phase: Both sham and chABC spent significantly more time interacting with Stranger 2, demonstrating rescue of social memory in aged mice. (F) Social memory index showed no difference between the groups. (G) Representative heatmaps of social memory phase. (H) Two trial test; Interaction during learning and recall phases with familiar stimulus: Both sham and chABC mice showed reduction in interaction during recall phase, indicating rescue of impaired social memory in aged mice. (I) Percentage reduction in interaction time from learning to recall phase was similar between the two groups. (J) Object recognition test; Preference for object 1 and 2 during familiarization phase. (K) Discrimination index showed no difference across the groups, indicating rescue of object recognition memory in chABC mice. *n* = 6 per group, all data represented are mean ± SEM; ns, non‐significant, *p < 0.05, **p < 0.01, Student's *t*‐test and two‐way ANOVA with Sidak's multiple comparison test.

To explore whether abnormal CA2 PNNs in aged mice have a causal link to play in recognition memory deficits, we next degraded the PNNs specifically in the CA2 of aged mice using bilateral chABC administration. In the three‐chamber test, sociability remained intact in both sham‐treated control and chABC‐treated aged mice (Figure 4B,D) (interaction time (s), sham, stranger 1: 158.50 ± 20.04, empty cage: 52.94 ± 9.70, *p* = 0.006, *t*(5) = 4.53; chABC, stranger 1: 133.80 ± 29.24, empty cage: 47.28 ± 11.48; *p* = 0.027, *t*(5) = 3.10, paired *t*‐test). The sociability index showed no statistical difference between the groups (Figure 4C) (sociability index, sham: 0.53 ± 0.06, chABC: 0.41 ± 0.10; *p* = 0.318, *t*(10) = 1.05, unpaired *t*‐test). Notably, in the social memory phase, chABC‐treated aged mice showed significant increase in interaction time with novel stranger 2 mice compared to stranger 1 (Figure 4E,G) (interaction time (s), sham, stranger 1: 65.78 ± 11.20, stranger 2: 112.90 ± 12.59, *p* = 0.002, *t*(5) = 5.77; chABC, stranger 1: 59.36 ± 15.46, stranger 2: 102.50 ± 26.85; *p* = 0.046, *t*(5) = 2.63, paired *t*‐test). The social memory index in chABC‐treated aged mice was also comparable to the sham group (Figure 4F) (social memory index, sham: 0.28 ± 0.05, chABC: 0.22 ± 0.10; *p* = 0.563, *t*(10) = 0.60, unpaired *t*‐test).

We further performed the two‐trial social memory test, where the chABC‐treated aged mice exhibited reduced average interaction time in the recall phase, similar to that of the sham control (Figure 4H) (interaction time (s), sham, learning: 61.66 ± 5.27, recall: 27.53 ± 3.33, *p* = 0.002; chABC, learning: 59.19 ± 9.75, recall: 33.38 ± 5.17, *p* = 0.018, two‐way ANOVA with Sidak's multiple comparison test). The percentage reduction in interaction time from learning to recall phases was similar among both groups (Figure 4I) (% reduction, sham: 54.69 ± 5.27, chABC: 48.75 ± 5.55; *p* = 0.455, *t*(10) = 0.78, unpaired *t*‐test). In summary, behavioral assessment of social recognition memory after chABC administration demonstrated that degrading excess PNNs in aged mice effectively rescued their social recognition memory deficits.

#### 3.4.2 PNN Depletion Rescued Deficits in Object Recognition Memory in Aged Mice

Further, we tested whether reducing PNN in CA2 affected object recognition by performing the NOR test. Both sham and chABC‐treated aged mice displayed similar preference for objects 1 and 2 during familiarization (Figure 4J, Figure S2H) (No. of approaches: sham, object 1: 50.90 ± 2.56, object 2: 49.10 ± 2.56, *p* = 0.740, *t*(5) = 0.35; chABC, object 1: 49.82 ± 1.80, object 2: 50.18 ± 1.80, *p* = 0.925, *t*(5) = 0.10; Interaction time (s), sham, object 1: 49.06 ± 1.09, object 2: 50.94 ± 1.09, *p* = 0.98; chABC, object 1: 47.45 ± 2.12, object 2: 52.55 ± 2.12, *p* = 0.57, paired *t*‐test). Interestingly, chABC administration restored the capability of aged mice to differentiate novel versus familiar objects, demonstrated by a discriminative index comparable to that of sham controls (Figure 4K, Figure S2I) (DI with number of approaches, sham: 0.24 ± 0.06, chABC: 0.18 ± 0.05, *p* = 0.432, *t*(10) = 0.82; DI with interaction time (s), sham: 0.59 ± 0.05, chABC: 0.47 ± 0.12, *p* = 0.797, unpaired *t*‐test).

### 3.5 PNN Depletion Improved Hippocampal Theta Oscillations in Aged Mice

To assess whether PNNs directly contribute to the attenuated theta oscillations observed, we investigated whether reducing PNNs could restore theta oscillatory activity. Power spectral analysis of LFP recording from the dCA2 of chABC‐treated aged mice across the 3–7 Hz theta range showed a notable increase in theta power in comparison to aged mice (Figure 3D). Further, an increase in the mean theta power and amplitude were observed in the chABC group (Figure 3E,F) (mean theta power, aged vs. chABC, *p* = 0.016; mean theta amplitude, aged vs. chABC, *p* = 0.003, one‐way ANOVA, post hoc Tukey–Kramer test). Interestingly, we also observed a shift in the theta frequency towards lower ranges in aged mice after chABC administration (Figure 3G) (peak frequency, aged vs. chABC, *p* = 0.015, one‐way ANOVA with post hoc Tukey–Kramer test).

### 3.6 ChABC Administration Restored PNNs to Control Level

After behavioral testing and electrophysiological recording, randomly selected mice from the chABC‐treated group were sacrificed for IHC. Our results revealed that WFA staining intensity and PNN density were restored to control levels in these mice (Figure 2B–D) (mean intensity: aged vs. chABC, *p* = 0.027; density (PNNs/mm^2^): aged vs. chABC, *p* = 0.012, one‐way ANOVA with post hoc Tukey–Kramer test).

## 4 Discussion

The capacity to recognize previously encountered stimuli, such as familiar individuals, objects, or locations, is a fundamental cognitive process critical for adaptive behavior and effective responses to environmental cues (Manns et al. 2003). However, the exact nature and underlying mechanism of recognition memory deficits in aging‐associated neurological disorders remain elusive. In the present study, we asked whether PNNs are involved in the regulation of hippocampal‐dependent memory and rhythmicity during aging and whether manipulation of PNNs can restore these functions.

Our findings that area CA2 of aged mice demonstrate a marked increase in WFA staining intensity are consistent with previous findings that have reported elevated PNNs in the CA2 of mouse models exhibiting social dysfunction (Carstens et al. 2021; Cope et al. 2022). Notably, we observed a similar pattern of increased WFA intensity in the CA2 of naturally aged mice as well. While a modest increase in WFA intensity was observed in CA1 and CA3 of both D‐gal and aged mice, this increase was more pronounced in CA2. Nonetheless, minor effects on surrounding hippocampal regions cannot be entirely ruled out. More broadly, PNN changes in the aging brain appear to be context specific. While increased PNN has been reported in the sensory cortex (Ueno et al. 2018), perirhinal cortex (Romberg et al. 2013; Yang et al. 2015), and inferior colliculus (Mafi et al. 2020), a decrease is observed in the prefrontal and ventral orbital cortex during aging (Ueno et al. 2023), and in the CA2 of an AD mouse model (Rey et al. 2022). These region‐specific differences are likely influenced by neuronal activity patterns and pathological conditions. Interestingly, while ChABC injection into the CA2 impaired social memory in wild‐type (WT) mice (Cope et al. 2022; Domínguez et al. 2019), the same treatment rescued social memory deficits in BTBR mice that exhibit elevated PNN levels (Cope et al. 2022), suggesting that optimal PNN levels are essential for maintaining the delicate balance between synaptic plasticity and stability. Disruption of this balance, whether through excessive accumulation or degradation, can significantly impact neuronal processes.

Functional changes to the PNN structure, specifically the sulfation patterns of CS‐GAGs, further underscore their role in synaptic plasticity. Sulfation changes in CS‐GAGs during aging are complex and involve multiple CS isomers. Sulfation patterns such as CS‐O (0S), CS‐D (2S6S), and CS‐E (4S6S) have been implicated in diffusion properties, neuroregeneration, and neuroinflammation, respectively (Gilbert et al. 2005; Shida et al. 2019; Syková and Nicholson 2008). However, in the context of our findings, we propose that the observed changes could be primarily influenced by CS‐4 and CS‐6 sulfation, given their well‐established roles in memory processes, neuroplasticity, and synaptic stability, as well as the potential of C4/C6 sulfation manipulation to restore cognitive deficits in age‐associated neurological disorders (Huang et al. 2023; Yang et al. 2017). Aging shifts the CS composition towards the less permissive C4‐sulfation, which can enhance the inhibitory properties of PNNs, creating a more restrictive environment for synaptic plasticity (Foscarin et al. 2017). Further, although WFA is widely used for labeling PNNs, it has been proposed that WFA primarily identifies PNNs with a 4‐sulfation pattern (Miyata and Kitagawa 2016). Thus, the increased WFA labeling observed in aged CA2 may reflect a shift towards an inhibitory 4‐sulfation pattern and/or an increase in the abundance of PNN components, though our current analysis does not allow us to distinguish between these possibilities. We further observed elevated gene expression of *Ptprz1* (Figure S2J), which encodes phosphacan, a CSPG critical for PNN structure and integrity, selectively in the dCA2 of aged mice. *Ptprz1* KO animals exhibit a loss of the reticular structure of PNNs despite most PNN components remaining neuron‐bound, suggesting that Ptprz1 is essential for the proper organization of PNNs, but not necessarily for the presence of individual components (Eill et al. 2020). In contrast, *Ptprz1* overexpression is implicated in conditions like schizophrenia, where altered PNN dynamics contribute to cognitive and synaptic dysfunction (Takahashi et al. 2011).

Interestingly, we observed that PNN attenuation in CA2 of aged mice was enough to rescue both social and object recognition memory, reflected by increased exploration of novel social and object stimuli. A recent study showed that C4‐sulfation regulates PNN density around CA2 pyramidal neurons, and its selective ablation restored social memory deficits (Huang et al. 2023). Similarly, restoration of the more permissive C6‐sulfation in aged mice has been shown to rescue object recognition memory (Yang et al. 2021). Moreover, PNN‐bearing PV+ neurons contribute to object memory, with new inhibitory inputs formed during memory acquisition. Aging increases the high‐expressing PV+ cells and decreases the low‐expressing PV+ neurons, a pattern shown to be reversed by ChABC treatment (Yang et al. 2021). This shift towards a low‐PV network state in the hippocampus after chABC likely amplifies inhibitory input to PV cells, leading to increased turnover of excitatory synapses and improved performance in novel object recognition.

A defining feature of the hippocampus is its prominent theta oscillations, which play a crucial role in correlating hippocampal activity to behavioral outcomes by providing a temporal framework for coordinating and integrating neural processes (Mehak et al. 2022). Disrupting PNNs, as shown in TN‐R knockout and chABC treatments, alters theta oscillation characteristics, including shifts in frequency and changes in power (Christensen et al. 2021; Gurevicius et al. 2004). Further, PNN attenuation in the CA2 has been shown to increase the excitability of fast‐spiking interneurons (FSIs) and alter their inhibitory interactions while increasing the pyramidal cell excitability (Hayani et al. 2018). In addition to their functions associated with inhibitory interneurons, PNNs within CA2 have a specific role in inhibiting synaptic potentiation at excitatory synapses on pyramidal neurons (Carstens et al. 2016). The interplay between PNNs and theta oscillatory dynamics during aging, however, is poorly understood. Elevated PNNs observed in the present study likely impair the synchrony of pyramidal and PV neuron activity, disrupting theta oscillations and, consequently, recognition memory deficits in aged mice. Enzymatic degradation of PNNs with ChABC restored theta power and recognition memory, suggesting that PNN‐mediated rigidity is a reversible contributor to cognitive decline. Notably, we observed a shift in theta frequency towards lower ranges following ChABC treatment, aligning with findings from a study reporting reduced peak frequency in TN‐R knockout mice with attenuated PNNs (Gurevicius et al. 2004). PNN degradation in aged CA2 possibly reduced the inhibitory C4S patterns, restoring neuronal flexibility and E/I balance. This may counteract the PNN‐induced stability that impairs theta‐modulated activity, thereby reinstating CA2 network dynamics and mitigating social recognition and novelty detection deficits.

While ChABC treatment is widely used to degrade PNNs, it is important to acknowledge that it also affects other ECM components beyond PNNs, including diffuse perisynaptic and axonal ECM. Although our injections were localized to the PNN‐rich CA2 region, we cannot fully exclude contributions from other ECM components to the observed functional outcomes, as diffuse perisynaptic ECM has also shown to regulate plasticity and memory consolidation (Chelini et al. 2024; Dankovich and Rizzoli 2022). Additional studies characterizing non‐PNN ECM components and their specific roles would help clarify their potential contributions to the observed effects. Furthermore, ChABC lacks the ability to differentiate between PNNs on CA2 pyramidal neurons and PV‐expressing interneurons, limiting insights into their cell‐specific roles. Additionally, WFA staining, while commonly used to visualize PNNs, is not selective and may also label diffuse ECM structures, which poses a limitation in distinguishing PNN‐specific changes. More targeted approaches, such as cell‐type‐specific genetic deletion of PNN components in either CA2 pyramidal neurons or PV interneurons, could help overcome this limitation. A recent study demonstrated that by conditionally deleting the *Acan* gene in either CA2 pyramidal neurons or PV‐expressing interneurons, distinct behavioral outcomes could be observed, with CA2 PNNs being important for social and spatial memory, while PV PNNs were crucial for contextual fear memory (Alexander et al. 2025). Further, the molecular mechanisms contributing to the observed increase in PNN intensity, such as altered CS‐sulfation patterns or changes in PTPRZ1 expression, were not explored in detail in this study. Future investigations examining these factors will provide deeper insights into the specific pathways driving PNN modifications in aging and their potential impact on cognitive function.

Overall, the present study identifies CA2 PNNs to be a key contributor to age‐associated deficits in recognition memory and theta oscillations. While our findings suggest that PNN removal restores both CA2 theta oscillations and recognition memory, establishing a direct causal link between these outcomes requires further investigation with selective manipulations. The CA2's high baseline PNN density and resistance to plasticity make it particularly vulnerable to age‐related changes. The specific mechanisms are complex and involve multiple interacting factors, including PNN structure, CS‐GAG sulphation, and the interplay between pyramidal cells and interneurons. Further research is needed to fully elucidate these mechanisms and develop targeted interventions to address cognitive deficits associated with PNN dysfunction.

## 5 Conclusion

Our findings suggest that the elevated PNN density in the dCA2 not only represents a structural alteration but also contributes to functional dysregulation of hippocampal networks during aging. We also identify the dorsal CA2 as an important neural substrate for processing hippocampal‐dependent recognition memory, highlighting its role beyond social behaviors. These findings suggest that targeting CA2 PNNs offers a promising avenue for therapeutic intervention to combat age‐related cognitive decline, with implications for conditions like Alzheimer's disease and other neurodegenerative disorders.

## Author Contributions

G.G. conceptualized the manuscript. S.F.M. performed all behavioral, electrophysiological, and molecular experiments. A.G. performed electrophysiological analysis. G.G., S.F.M., and A.G. wrote and approved the manuscript. A.B.S., F.J., and V.G.P. performed review and editing.

## Ethics Statement

The authors have reviewed the paper and approved of the content and this manuscript. The authors confirm that this manuscript has not been submitted for publication elsewhere. The authors affirm that the data in this manuscript are original. All the experiments carried out in this study were approved by the Institutional Animal Ethical Committee (IAEC), Kasturba Medical College (KMC), Manipal Academy of Higher Education (MAHE), Manipal, India.

## Conflicts of Interest

The authors declare that the research was conducted in the absence of any commercial or financial relationships that could be construed as a potential conflicts of interest. Figures were created with BioRender (https://www.biorender.com/).

## Supporting information


Data S1.

## Data Availability

Data will be made available on request.
