# Supplementary material for: Targeting CA2 Perineuronal Nets Restores Recognition Memory and Theta Oscillations in Aged Mice
Source: Aging Cell. 2025 Jun 16;24(9):e70139. doi: 10.1111/acel.70139 (PMC12419842; doi:10.1111/acel.70139)
Supplement: Supplementary file 1 — Data S1. [file ACEL-24-e70139-s001.docx]

**Supplementary information**

**Targeting CA2 Perineuronal Nets Restores Recognition Memory and Theta Oscillations in Aged Mice**

Sonam Fathima Mehak, Apoorva Bettagere Shivakumar, Feyba Jijimon, Amritanshu Gupta, Vikram Gopalakrishna Pillai, Gireesh Gangadharan

**Supplementary methods**

**Assessment of locomotion and anxiety-like behavior**

***Open field test***

To assess potential differences in locomotion that might affect performance in behavioral assays, mice underwent the open field test. Briefly, they were placed in a 40 x 40 x 40 cm open-field arena and allowed to explore freely for 30 minutes (Zhang et al., 2016). Total distance traveled was measured to evaluate locomotor activity, while entries into the center of the arena were used to gauge anxiety levels. The cumulative distance traveled and distance covered in 5-minute intervals were quantified.

***Elevated plus maze***

The apparatus was a plus-shaped maze with two opposite open arms (31 x 6 cm) and two closed arms of the same dimensions, enclosed by 15 cm high walls, all connected by a central square chamber. The maze was elevated 50 cm above the ground. Mice were placed in the central chamber facing an open arm and allowed to explore for 5 minutes. The time spent in open and closed arms, total distance traveled, and percentage of entries into open arms were recorded. The protocol was adapted from Walf & Frye, (2007).

**Quantitative real-time polymerase chain reaction ((qRT-PCR)**

Mice were euthanized via cervical dislocation and brain samples were collected. The hippocampus was coronally dissected using an ice-cold brain matrix (SA2165, Roboz, USA), and dorsal CA2 isolated from the surrounding tissue with the help of a scalpel. After total RNA extraction using TRIzol (TR118200, Molecular Research Centre, USA), cDNA was subsequently synthesized using 2 μg of the extracted RNA with the high-capacity cDNA reverse transcription kit (Applied Biosystems, Thermo Fisher Scientific, USA). qRT-PCR was performed for the *Ptprz1* (forward primer: GGGATTTGGTAGTGAGTCTG, reverse primer: CCCTCAACGGTATAAGAAGG) gene with *B-actin* as the internal control. Reactions were conducted in duplicates using the PowerUp SYBR Green Master Mix (Applied Biosystems, Thermo Fisher Scientific, USA) in a QuantStudio 6 Pro Real-Time PCR System (Applied Biosystems, Thermo Fisher Scientific, USA) according to the manufacturer’s instructions. The expression level of the gene of interest was evaluated using the 2−(ΔΔCt) method. The PCR product quality was monitored using post-PCR melt-curve analysis at the end of the amplification cycles.

**Supplementary results**

**Aged mice exhibit intact locomotion and no anxiety-like behavior**

Overall locomotory activity and anxiety levels were measured using a 30-minute open field test. Both the control and aged mice exhibited comparable levels of locomotion (**Supplementary figure S2A**), with similar distance traveled in each 5-minute interval (interaction: F(5,60)=1.25, two-way ANOVA) (**Supplementary figure S2B**). The cumulative distance traveled over the 30-minute session also showed no significant difference (distance traveled (cm): control, 6797 ± 741.30; aged, 8595 ± 664.40, p=0.101, t(10)=1.81, unpaired *t*-test) (**Supplementary figure S2C**). Furthermore, the frequency of entries to the center zone of the open field was similar between the control and aged mice (control: 46.83 ± 10.36, aged: 41.83 ± 9.56, p=0.730, t(10)=0.35, unpaired *t*-test) (**Supplementary figure S2D**), suggesting no changes in anxiety-like behavior.

Changes in anxiety-like behavior were further confirmed using the elevated plus maze (**Supplementary figure S2E**), where the percentage of entries to open arms (saline: 43.19 ± 4.58, D-gal: 33.90 ± 5.07, p=0.203, t(10)=1.36, unpaired *t*-test) (**Supplementary figure S2F**) and total distance traveled (distance traveled (cm): control, 1954 ± 123.40; aged, 1603 ± 110.70, p=0.060, t(10)=2.12, unpaired *t*-test) (**Supplementary figure S2G**) were observed to be similar in both aged and control mice. These findings indicate that the observed behavioral changes were not influenced by motor or anxiety-related factors.

**Increased *Ptprz1* gene expression in CA2 of aged mice**

The Ptprz1 gene (Protein Tyrosine Phosphatase Receptor Type Z1) encodes receptor-type protein tyrosine phosphatase, or phosphocan, that is involved in several neuronal processes and plays a role in brain plasticity. Studies have shown that PTP is predominantly expressed in PNN-wrapped neurons, where PTPRZ1 regulates the assembly and degradation of CSPGs in the extracellular matrix through its phosphatase activity, impacting the structural integrity and density of PNNs (Eill et al., 2020). Our gene expression data revealed that *Ptprz1* is significantly upregulated in the CA2 of aged mice in comparison to control (3.94 ± 1.00, normalized to control, p=0.042, t(4)=2.94, unpaired *t*-test) (**Supplementary figure S1J**), suggesting an age-related enhancement in PTPRZ1 activity, potentially contributing to altered PNN dynamics.

Eill, G. J., Sinha, A., Morawski, M., Viapiano, M. S., & Matthews, R. T. (2020). The protein tyrosine phosphatase RPTPζ/phosphacan is critical for perineuronal net structure. *The Journal of Biological Chemistry*, *295*(4), 955–968. https://doi.org/10.1074/jbc.RA119.010830

Walf, A. A., & Frye, C. A. (2007). The use of the elevated plus maze as an assay of anxiety-related behavior in rodents. *Nature Protocols*, *2*(2), 322–328. https://doi.org/10.1038/nprot.2007.44

Zhang, J.-B., Chen, L., Lv, Z.-M., Niu, X.-Y., Shao, C.-C., Zhang, C., Pruski, M., Huang, Y., Qi, C.-C., Song, N.-N., Lang, B., & Ding, Y.-Q. (2016). Oxytocin is implicated in social memory deficits induced by early sensory deprivation in mice. *Molecular Brain*, *9*(1), 98. https://doi.org/10.1186/s13041-016-0278-3

**Supplementary figures and legends**

**Supplementary figure S1**

**
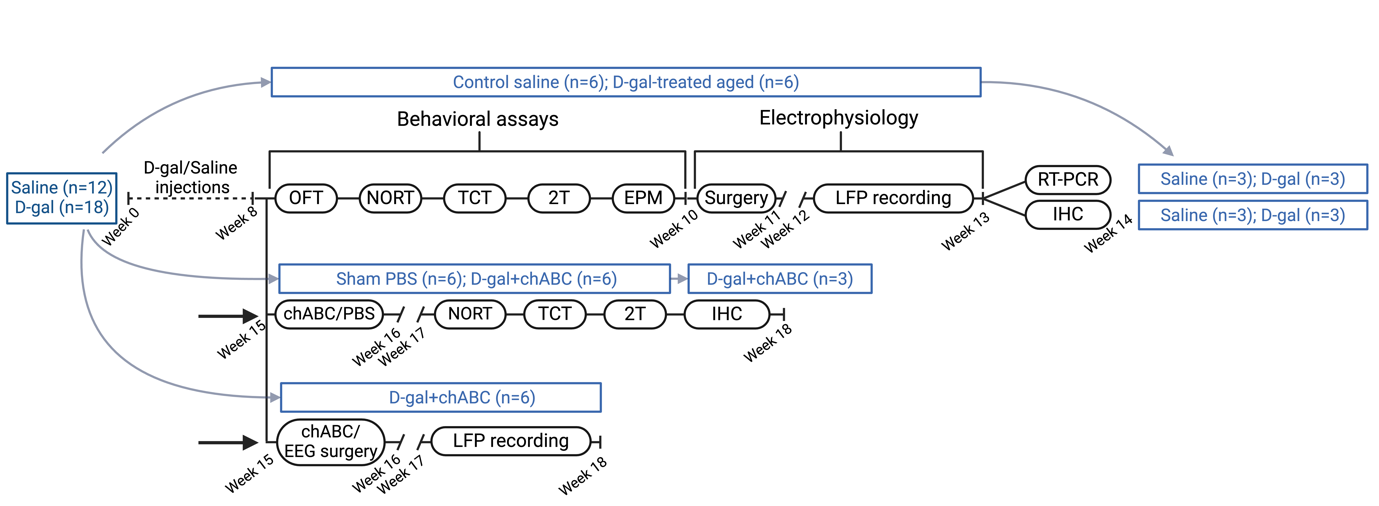
**

**Figure S1: Timeline of the experiments**

**Supplementary figure S2**

**
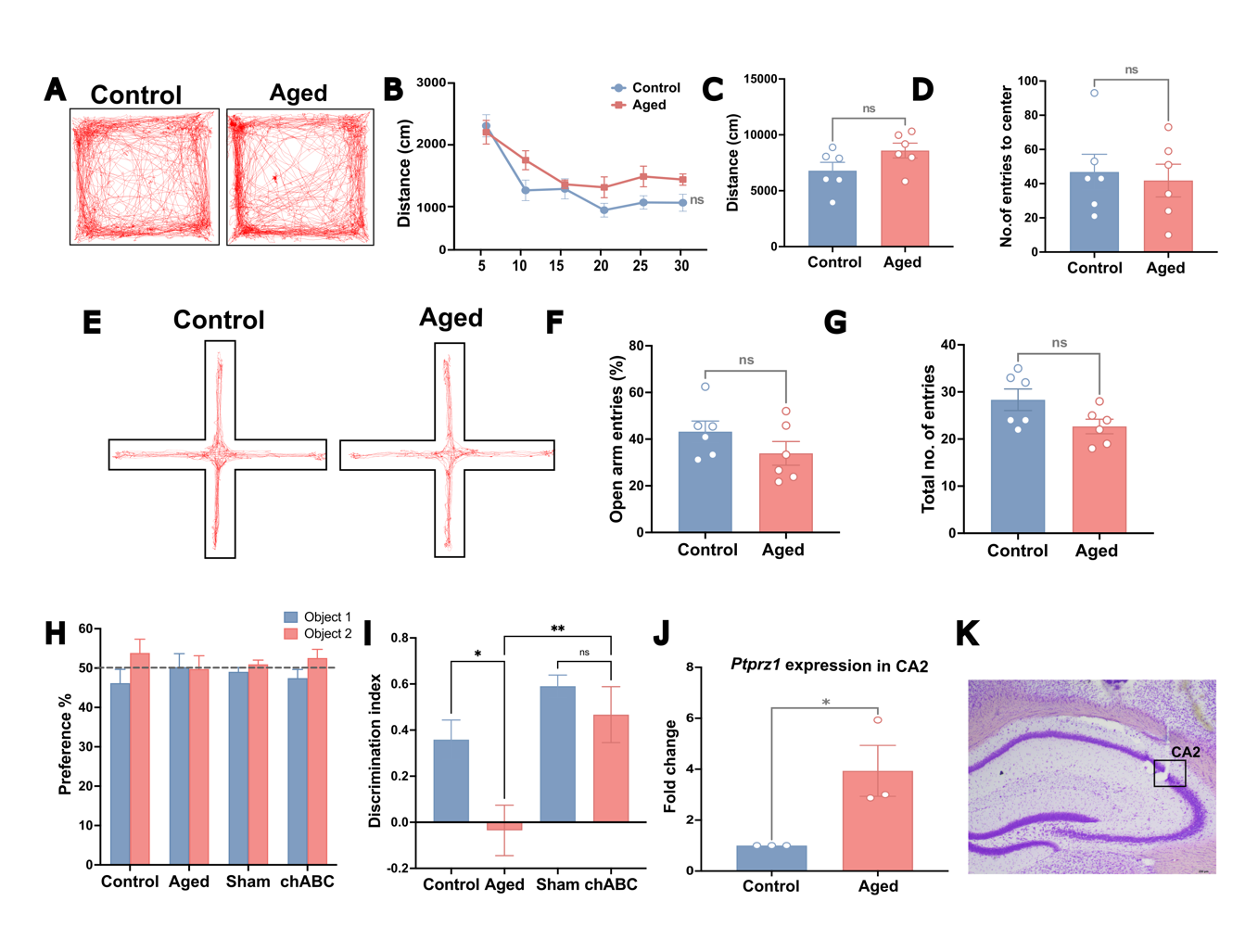
**

**Figure S2: (A-D) Intact locomotion in aged mice.** (A) Representative trackplots showing movement trajectories of control and aged mice. (B) Distance travelled (cm) in five minute bins. (C) Total distance travelled. (D) Number of entries to the center of the open field**. (E-G) Unaltered anxiety-like behavior in aged mice**. (E) Representative trackplots showing movement trajectories of control and aged mice. (F) Percentage (%) of open arm entries (G) Total number of entries to both arms. **(H&I) Reduced interaction (s) with novel object in NOR**. (H) Percentage preference for object 1 and object 2 in familiarization phase. (I) Reduced discrimination index displayed aged group, rescued by chABC. (J) Increased gene expression of *Ptprz1* in the CA2 of aged mice. (K) Representative Nissl-stained coronal section showing electrode placement for LFP recording.


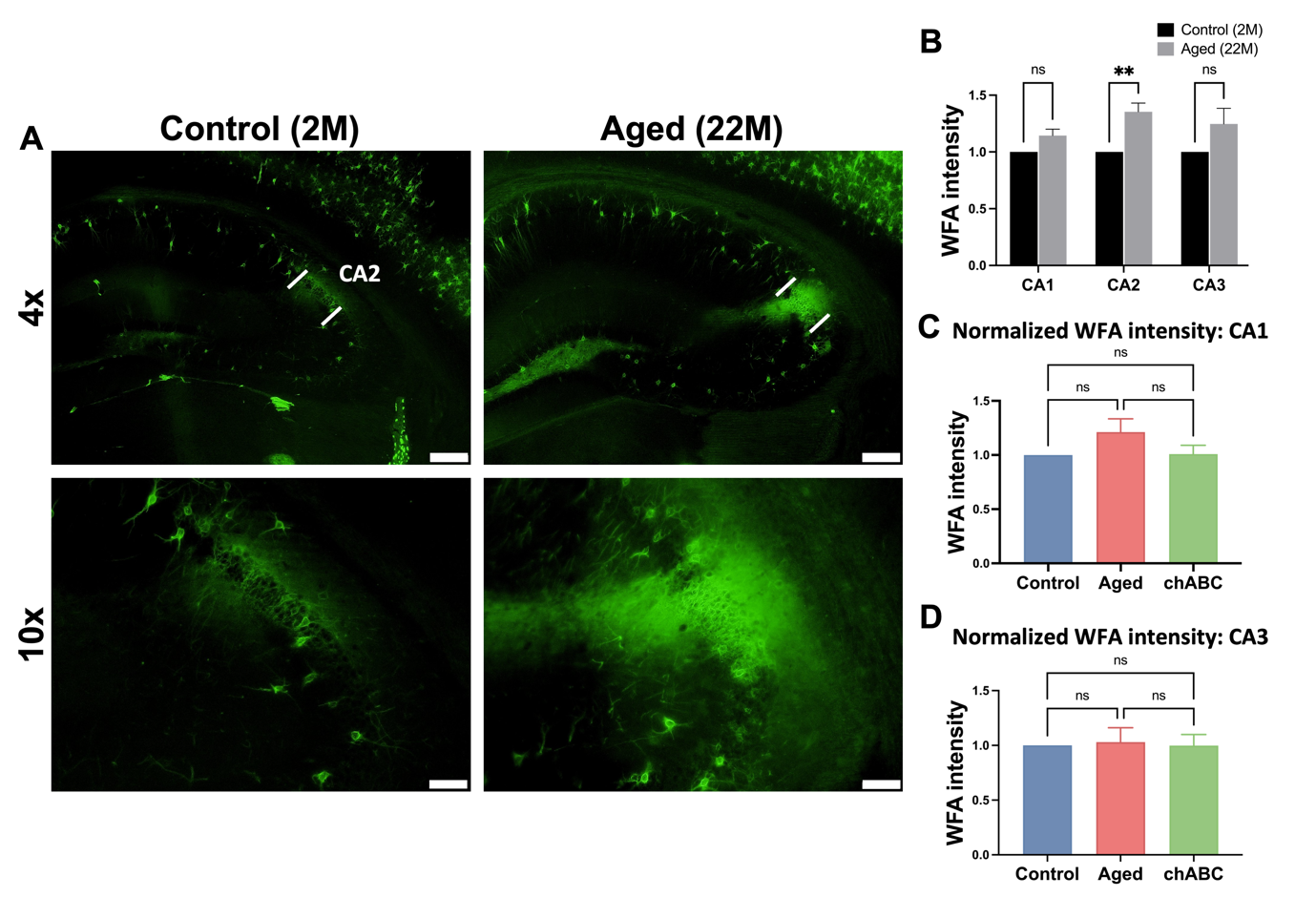


**Figure S3: (**A) Naturally aged mice (22M) show increased WFA fluorescence intensity in the dCA2. (B) Normalised WFA intensity in the CA1, CA2, and CA3 of aged mice (Mean intensity, control versus aged, CA1 p=0.40, CA2 p=0.009, CA3 p =0.07, one-way ANOVA with post-hoc Tukey-Kramer test). Normalised WFA intensity in the (C) CA1 and (D) CA3 of aged (D-gal) mice showing no difference between the groups. (Mean intensity CA1: control versus aged, p=0.201, CA3: control versus aged, p=0.972, one-way ANOVA with post-hoc Tukey-Kramer test).
